# Digital Marketing of Breast-Milk Substitutes: a Systematic Scoping Review

**DOI:** 10.1007/s13668-022-00414-3

**Published:** 2022-05-04

**Authors:** Alexandra Jones, Soumyadeep Bhaumik, Georgia Morelli, Jing Zhao, Miranda Hendry, Laurence Grummer-Strawn, Nina Chad

**Affiliations:** 1 Food Policy Division, The George Institute for Global Health, UNSW, Level 5, 1 King St, Newtown, NSW 2042 Australia; 2 Meta-Research and Evidence Synthesis Unit, The George Institute for Global Health, India, 308, Elegance Tower, Plot No. 8, Jasola District Centre, New Delhi, 110025 India; 3 School of Public Health, University of Sydney, Edward Ford Building, A27 Fisher Rd, Sydney, NSW 2006 Australia; 4 Actions in Health Systems Unit, Department of Nutrition and Food Safety, World Health Organization, Avenue Appia, 20, Ch-1211, 27 Geneva, Switzerland

**Keywords:** Breast-milk substitutes, Infant formula, Breastfeeding, Marketing, Digital, Social media, Commercial milk formula, International Code of Marketing of Breast-milk Substitutes, Health promotion, Infant nutrition, Young child feeding

## Abstract

***Purpose of Review*:**

Globally, too few children are breastfed as recommended. Commercial promotion of breast-milk substitutes (BMS) is one factor undermining breastfeeding globally. Although the International Code of Marketing of BMS prohibits all forms of marketing, promotion has been observed in digital environments. We aimed to understand the scope and impact of digital marketing for the promotion of BMS.

***Recent Findings*:**

BMS are promoted strategically and in an integrated fashion across multiple digital channels (social media, manufacturer websites, online retailers, blogs, mobile apps and digital streaming services). Traditional marketing strategies like gifts, discounts and coupons are also disseminated digitally. Data mining, real-time direct-to-consumer advertising and partnering with peer-group social media influencers are additional avenues. Exposure to digital marketing is common. Research on the impact of digital marketing is scarce, but its negative impact on breastfeeding intention and initiation has been documented. Case reports from marketing industry press corroborate academic evidence by highlighting the benefits of digital marketing to BMS companies in recruiting new users and increasing sales.

***Summary*:**

To protect and promote breastfeeding, coordinated global action and strengthened national measures will be needed to implement, monitor and enforce the International Code in a digital context. Further action could include voluntary restrictions on BMS marketing by social media platforms and greater use of government-led data and health privacy regulation.

## Introduction

Although the World Health Organization (WHO) recommends that infants be fed only breast-milk for the first 6 months of life, fewer than 45% of infants under 6 months of age were exclusively breastfed between 2014 and 2021 [1]. Promotion of breast-milk substitutes (BMS) undermines efforts to improve breastfeeding practices globally [2]. The International Code of Marketing of Breast-milk Substitutes 1981 (the Code) aims “to contribute to the provision of safe and adequate nutrition for infants, by the protection and promotion of breastfeeding, and by ensuring the proper use of BMS, when these are necessary, on the basis of adequate information and through appropriate marketing and distribution” [3]. The Code calls upon governments to enact domestic legislation to regulate the marketing of BMS. While progress has been made in implementing the Code at country level [4, 5], challenges to full and effective implementation persist [5].

In 2001, the World Health Assembly (WHA) expressed concern over the use of “new modern communication methods, including electronic means” to promote products within the scope of the Code and called on governments to strengthen mechanisms to ensure compliance in all forms of media [6]. Digital marketing practices that were not yet conceived when the Code was written are now commonplace [2].

In 2020, the seventy-third WHA requested that the WHO Director General prepare a comprehensive report to understand the scope and impact of digital marketing strategies for promotion of BMS for the seventy-fifth WHA in 2022 [7]. We conducted a systematic review of published literature to inform this work.

## Methods

A systematic scoping review approach (registration https://osf.io/5ftxp/) was used. Studies published in English since the 1st January 2000 were included if they reported one of the following outcomes: content of digital marketing of BMS; exposure to digital marketing of BMS; knowledge, attitudes and beliefs about BMS; intention to use BMS; and behaviours including uptake/initiation of BMS use, frequency/intensity/quantity of BMS use, continuation of BMS use, stopping exclusive breastfeeding, continuation of breastfeeding or stopping breastfeeding.

Marketing was defined as “the activity, set of institutions, and processes for creating, communicating, delivering and exchanging offers that have value for customers, clients, partners and society at large”, using the definition of the American Marketing Association [8]. Digital marketing was defined as any form of marketing that used a digital channel or platform. We adopted an inclusive definition of digital channels and platforms that included, but was not limited to, social media, search engines, display advertising, digital broadcast, company or third-party websites, streaming services, direct channels (e.g. SMS, email) and smart phone apps. “Breast-milk substitutes” were defined as “any milks (or products that could be used to replace milk, such as fortified soy milk), in either liquid or powdered form, that are specifically marketed for feeding infants and young children up to the age of 3 years, including follow-up formula and growing-up milks” [9, 10].

Five academic databases (PubMed, MEDLINE, Embase, APA PsycINFO, Cochrane Register of Controlled Trials) were searched and the World Advertising Resource Centre (WARC) database, which includes case reports of award-winning marketing campaigns submitted by marketing companies. We also hand-searched websites of organisations known to advocate for the implementation of the Code to identify relevant grey literature. For a list of search terms and websites searched see the Appendix.

Two authors (J. Z. and M. H.) independently screened each record based on titles and/or abstracts and marked each record as “exclude” or “include”. Disagreements, if any, were resolved by discussion with a third author (A. J.) being an adjudicator. Full texts of “included” studies were assessed against the eligibility criteria and disagreements, if any, were handled similarly.

At least two authors (J. Z. and G. M.) extracted data independently using a piloted data extraction form. Disagreements were resolved through consensus discussions. No critical appraisal of included studies was conducted.

As there are no globally accepted typographies of digital marketing strategies available, we used a self-adaptive narrative synthesis approach to the analysis [11] and organised the results thematically.

Marketing industry case reports were treated separately in our synthesis (post hoc decision) because they did not describe study methodology in detail, and the outcomes reported were not readily comparable with other publications.

## Results

**Fig. 1:**
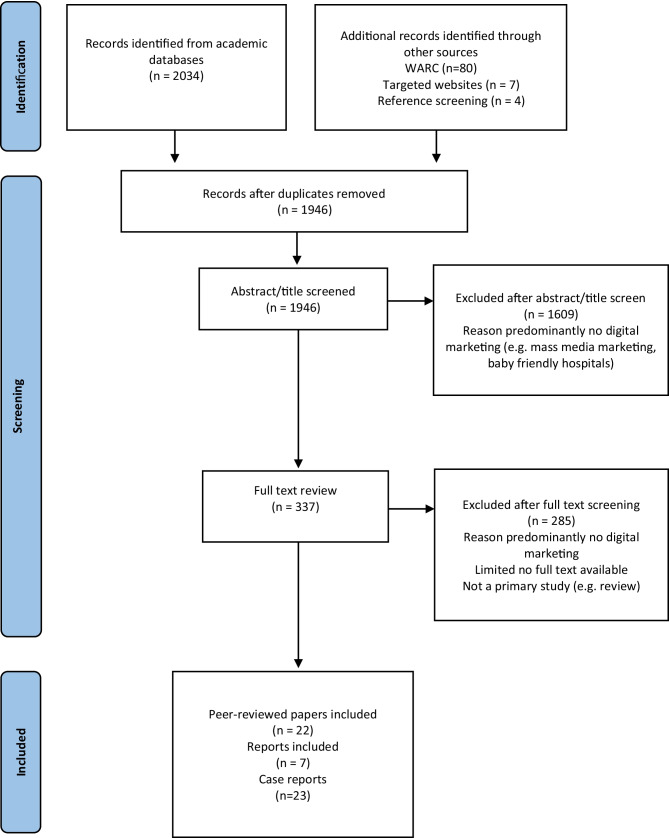
Flow chart of studies and case reports through the review process

We initially identified 2097 records from academic databases and seven documents from hand-searches. After screening, 29 studies were identified that met the eligibility criteria (Fig. 1 PRISMA flowchart). Characteristics of included studies are presented in Table 1.

**Table 1:** Summary of the included publications (peer-reviewed studies and independently published reports)

**Author (year of publication)**	**Study design**	**Study setting**	**Study category (scope/impact)**	**Study population**	**Digital marketing channel or platform**	**Digital marketing strategies used**
Abrahams (2012) [12]	Content analysis	USA	Scope	NA	Social media (Facebook, MySpace, Twitter, YouTube, Google +), blogs, mobile applications, interactive websites	Initiating direct contact with mothers Providing educational material Offering coupons, discounts, free samples and gifts Use of health or nutrition claims and/or images and text idealising infant formula
Bartolini et al. (2009) [42]	Cross-sectional study	Brazil	Scope	NA	Websites	Use of health or nutrition claims and/or images and text that idealise formula feeding/absence of required statements
Bass (2002) [37]	Qualitative study	UK	Scope	NA	Manufacturer websites	Offering coupons, discounts, free samples and gifts
Berry and Gribble (2017) [41]	Content analysis	Australia	Scope	NA	Websites	Use of health or nutrition claims and/or images and text idealising infant formula
Brewer et al. (2020) [27]	Policy analysis	Indonesia, India, Nigeria, Vietnam, Thailand	Scope	NA	Online retail	Offering coupons, discounts, free samples and gifts
Changing Markets Foundation (2017) [22]	Mixed-methods	World	Scope	NA	Social media, sponsored blogs, mobile applications, personalised emails	Collecting consumer data Influencer marketing
Ching et al. (2021) [25•]	Mixed-methods study	World	Scope	NA	Social media (Facebook, Instagram), online shopping portal, partner-NGO website, online news portal, company website, public health website blog	Providing educational material Offering coupons, discounts, free samples and gifts
Davis et al. (2020) [34]	Content analysis	USA	Scope	NA	Blogs	Use of health or nutrition claims and/or images and text idealising infant formula
Department of Health Hong Kong (2013) [26]	Cross-sectional survey	Hong Kong	Scope/impact	Mothers with singleton pregnancy; Cantonese speaking, lived in Hong Kong for > 1 year prior to survey	Manufacturer websites, social media, online forums, electronic ads, email messages	Providing educational material
Global Breastfeeding Collective (2020) [20]	Policy analysis	World	Scope	NA	Social media (Facebook), sponsored blogs, vlogs, online magazines	Influencer marketing Offering coupons, discounts, free samples and gifts
Gunter et al. (2013) [38]	Content analysis	UK	Scope	NA	Manufacturer websites	None
Harris et al. (2017) [21]	Content analysis	USA	Scope	NA	Social media, sponsored blogs, manufacturer websites	Influencer marketing Offering coupons, discounts, free samples and gifts Use of health or nutrition claims and/or images and text idealising infant formula
Hastings et al. (2020) [28]	Mixed-methods study	UK, Europe, North America, Australia, New Zealand	Scope	Industry experts and professionals with experience of marketing BMS	Mobile applications	Collecting consumer data
Huang et al. (2013) [57]	Cohort study (national longitudinal study)	USA	Impact	Mothers aged > 18; single births; neither mother nor infant had a health condition likely to affect feeding	Internet	Providing educational material on infant and young child feeding
IBFAN (2018) [36]	Policy analysis	China, India, Indonesia, Mongolia, Philippines, Korea, Sri Lanka, Thailand	Scope	NA	Manufacturer websites, retail websites	Offering coupons, discounts, free samples and gifts
Jaichuen et al. (2018) [49]	Cross-sectional study	Thailand	Scope	NA	Digital TV	Use of images and text that idealise formula feeding
Lozada-Tequeanes et al. (2020) [16]	Cross-sectional study	Mexico	Scope	NA	Social media (Twitter, Facebook, YouTube)	Initiating direct contact with others Use of health or nutrition claims and/or images and text idealising infant formula
Mak (2015) [17]	Mixed-methods study	Hong Kong	Scope	Couples who had at least one child aged 3 years or younger	Blogs, social media (Facebook), parenting websites	Influencer marketing
Masin et al. (2018) [19•]	Qualitative study	World	Scope	NA	Social media (Twitter, Facebook, Instagram), mobile messaging, sponsored blogs, mobile applications	Offering coupons, discounts, free samples and gifts
Mejia et al. (2016) [15]	Content analysis	USA	Scope	NA	Social media (Facebook, Twitter), blogs	Influencer marketing Initiating direct contact with mothers
Newby et al. (2015) [40]	Cohort study	Australia	Scope	Healthy women aged 18 years and over, first time mothers	Retailer websites, parenting websites	Providing educational material on infant and young child feeding
Pereira-Kotze et al. (2020) [23•]	Policy analysis	South Africa	Scope	NA	Social media (Facebook, Instagram)	Initiating direct contact with mothers
Prado and Rinaldi (2020) [39]	Cross-sectional study	Brazil	Scope	NA	Manufacturer websites	Use of health or nutrition claims and/or images and text idealising infant formula
Senkal and Yildiz (2019) [13]	Content analysis	Europe	Scope	NA	Social media (Facebook, Instagram, Twitter), blogs, mobile applications, interactive websites	Initiating direct contact with mothers Influencer marketing Use of health or nutrition claims and/or images and text idealising infant formula
Vinje et al. (2017) [14]	Content analysis	Cambodia, Indonesia, Myanmar, Thailand and Vietnam	Scope	NA	Social media (Facebook posts or conversations)	Initiating direct contact with mothers Influencer marketing
Walker (2012) [18]	Qualitative study	World	Scope	NA	Social media (Facebook, Twitter), mobile applications, websites	Offering coupons, discounts, free samples and gifts
Wilking (2020) [24••]	Policy analysis	USA	Scope	NA	Social media, websites, influencers, digital display ads, banner ads, email messages, purchase reminders	Collecting consumer data Offering coupons, discounts, free samples and gifts
Zhang et al. (2013) [56]	Cohort study (national longitudinal study)	USA	Impact	Healthy mothers with healthy term or near-term singleton infants	Websites	Providing educational material on infant and young child feeding
Zhao et al. (2019) [29]	Content analysis	China	Scope	NA	Mobile applications	Offering coupons, discounts, free samples and gifts Use of health or nutrition claims and/or images and text idealising infant formula

Our search of the WARC database returned 80 records. From these, we identified 22 case reports for inclusion. One additional case report was identified from the references of included studies. Characteristics of included case reports are presented in Table 2.

**Table 2:** Summary of included World Advertising Research Centre Database case reports

**Manufacturer name (year)**	**Country**	**Digital marketing channel/platform**	**Strategies used**	**Reported business impact**
Abbott Laboratories Philippines (2013) [48]	Philippines	Manufacturer website	Providing educational material on infant and young child feeding	20% increase in market share (previous year was 12%)
Abbott Nutrition Malaysia (2019) [45]	Malaysia	Social media (Facebook)	Initiating direct contact with mothers	E-commerce site became the bestselling storefront in its category on Lazada by end of 2017
Danone-Nutrition (2014) [46]	China	Internet	Collecting consumer data	Gained incremental market share and brand preference
Danone-Nutrition (2017) [55]	Indonesia	Social media (YouTube, Facebook, Google + , InMobi)	Collecting consumer data Offering coupons, discounts, free samples and gifts	Reached over 1 million mums
Fonterra Brands Malaysia (2012) [35]	Malaysia	Social media, sponsored blogs, website	Influencer marketing	200,000 mothers switched to product, garnered a 2.4% market share increase in 3 months
FrieslandCampina Friso (2017) [32]	Vietnam	Mobile messaging (Zalo)	Initiating direct contact with mothers Collecting consumer data	250 k USD was generated from new users and Friso’s Zalo account gained 18 k followers in two months
FrieslandCampina Friso (2018) [33]	Indonesia	Social media (WhatsApp)	Initiating direct contact with mothers	Achieved an increase of new users by 67% (compared to full year 2016) at 50% lower costs
FrieslandCampina Friso (2018) [47]	Vietnam	Social media (Facebook), e-commerce, online store	Initiating direct contact with mothers	Achieve a cost per new user of 20% lower compared to the previous year
FrieslandCampina Friso (2018) [52]	Vietnam	Social media (Facebook, Instagram, Zalo, Coc Coc, Bing, Google, GDN, Adtima), online store	Use of health or nutrition claims and/or images and text idealising infant formula	Increased sales and engagement
FrieslandCampina Friso (2011) [59]	Vietnam	Social media (Facebook), parenting forums	Initiating direct contact with mothers	More than 10,000 active Friso fans on Facebook, with more than 17,000 likes
Illuma Organic (2018) [44]	Hong Kong	Internet (Yahoo)	Providing educational content	Successfully cultivated 42,000 + audience
Mead Johnson (2018) [58]	USA	Social media (Facebook, Instagram)	Social media advertisements	A 27-point lift in ad recall; 15-point lift in preference; 14-pointlift in purchase intent
Nestlé (2016) [60]	India	Social media (YouTube)	Providing educational content	Brand boosted by 5.5 million YouTube views
Nestlé (2018) [61]	Singapore	Social media (Facebook)	Providing educational content Influencer marketing	Mother’s Day video achieved almost 100,000 views, over 400 shares and 100 comments, with engagement rates reaching 11%
Nestlé (2018) [50]	Mexico	Music-streaming service (Spotify)	Audio stories	Delivered more than 4.2 million impressions, gaining a unique reach of almost 500 k
Nestlé (2017) [62]	Singapore	Social media (Facebook)	Initiating direct contact with mothers	Built connection between the brand and targeted pregnant women
Nestlé (2017) [63]	Singapore	Social media (Facebook)	Influencer marketing	Over 200,000 video views and 700,000 impressions across multiple platforms
Nestlé (2017) [64]	Singapore	Social media (Facebook, Instagram)	Social media advertisements	Engagement rate peaked at 34%, with over 300,000 video views in 2 weeks
Nestlé (2010) [51]	China	Online video channel	Providing educational material	13 million video views in 3 months
Nestlé (2020) [54]	China	Internet	Collecting consumer data	Garnered 450 million impressions, increased sales by 32%
Wyeth Nutrition (2017) [30]	China	Mobile applications	None	Grew new users by 246% in one year
Wyeth Nutrition (2014) [31]	Hong Kong	Mobile applications	None	New user base grew by 65%, sales increased by 30%
Yili (2020) [53]	China	Artificial intelligence	Providing educational material	More than US$2.2 million in sales

### Scope of Digital Marketing of BMS

#### Social Media

We found evidence that BMS companies use social media to promote their brands and products [12–18, 19•, 20–22, 23•, 24••, 25•, 27] (Table 1), especially via Facebook, Twitter, Instagram and YouTube. BMS brands maintain accounts across multiple social media channels, and this appears to form part of a broader marketing strategy [12, 13, 15, 21, 24••, 25•].

BMS manufacturers adapt their marketing techniques to harness the functionality of social media platforms. Facebook was used to promote products on manufacturers’ own pages, for third-party advertising, and to foster peer-to-peer engagement [12–17, 19•, 20, 22, 23•, 24••, 25•]. Twitter is used to disseminate marketing messages and to notify users about promotions, such as photo contests, discounts and giveaways [12, 15, 18, 19•]. YouTube is used to disseminate video content, including advertisements [12, 16, 21, 22, 24••]. Instagram [13, 19•, 23•, 24••, 25•] is used to disseminate image-based promotions including some that idealise the use of BMS or depict infants less than 6 months old [13]. Case reports published in marketing industry press describe the power of social media engagement to drive sales (Table 2). Selected case studies of these campaigns are outlined in Box 1.

**Box 1:** Examples of BMS marketing campaigns from the WARC database

**Mead Johnson Enfamil targeted Facebook and Instagram advertising (2018)** [58]	
In the USA, Mead Johnson disseminated video and photo advertisements on Instagram and Facebook to increase awareness and drive purchase intent for its new infant formula product. Harnessing knowledge that “mothers often spend time, connect with other parents and discover new products on Instagram and Facebook”, the brand created content encouraging mothers who wanted to “reclaim the calm”, to *learn now* or *shop now* by clicking on a link to the brand’s website. Using a tool that identifies new mothers and analyses their responses to content placed on their newsfeeds, advertisements were delivered to either their Instagram or Facebook feeds depending on which was “most likely to drive the best campaign results at the lowest possible cost at any given time”. The 8-week campaign drove a 27-point increase in advertisement recall, a 15-point increase in preference for Mead Johnson products and a 14-point increase in intention to purchase Mead Johnson infant formula	
**Yili JinLingGuan (JLG) AI Baby Expert (2019–20)** [53]	
In 2019, Yili partnered with smart speaker manufacturer Xiaomi to create an artificial intelligence (AI) “baby expert”. The aim was to build the brand’s image among technically savvy young Chinese parents and drive sales. The speaker was loaded with > 1000 mum-and-baby-related questions and answers (Q&A), allowing parents to engage hands-free. Each answer ended with “answers provided by research experts at JLG”. Existing speaker owners were given access to Yili’s content, and 10,000 speakers were given to parents who purchased a large volume of product. Users made more than twice as many queries (> 55,000,000) and the campaign drove > USD 2.2 M in sales	
**FrieslandCampina Friso using messaging apps to recruit new users (2017)** [47]	
In Vietnam, Friso partnered with the number one national messaging app Zalo to more efficiently identify and reach new users. They used a database of potential consumers generated from data collected when individuals interacted with their online campaigns, e.g. on Facebook, and used an application programming interface to map whether these individuals were Zalo users. They found that 80–90% of Vietnamese mothers were already Zalo users, allowing them to utilise the app as a convenient communication method. They sent personalised messages inviting mothers to follow Friso’s Zalo account and followed up with prompts to validate their personal information on the Zalo app as well as choosing a time to receive a call from Friso’s call centre. Friso’s Zalo account gained 18,000 new followers in 2 months, 20% of user data was auto-validated saving human resource, and newly acquired users generated USD $250,000	
**Nestlé Excella Gold short stories on Spotify (2018)** [50]	
Nestlé produced 12 audio short stories “to development movement, communication skills and creativity” and created a branded Spotify profile to relaunch its Excella Gold formula range and increase brand awareness. Audio ads were played between songs on Spotify, and users were also invited to participate in “sponsored sessions” where they could earn 30-min uninterrupted streaming by watching a video advertisement for a BMS product. The campaign achieved > 4.2 million impressions and reached 500,000 mothers	

#### Mobile Applications

Free smart phone applications (apps) are used to promote BMS to parents [12, 13, 18, 19•, 22, 28, 29]. These apps offer parents’ general information on a range of parenting topics; infant feeding advice; tools for recording and sharing (their own or their baby’s) health information and personal reflections (journal entries); and weekly updates on baby development and provide platforms for engaging socially with other mothers or parents.

Apps also deliver BMS promotions. One paper analysed the content of 353 BMS advertisements that appeared on Chinese apps for parents [29]. Of the 79 advertisements published by 31 companies, 75 linked to e-shops, 39 were price promotions, 25 made product quality claims, and five included celebrity endorsements. Few of the advertisements included a direct advertisement disclosure [29].

BMS manufacturers create dedicated apps to reach parents with BMS promotions. We found reports of apps offering parents a tool for tracking babies’ consumption of BMS linked to an automatic formula milk preparation device [30], educational activities for young children [31] and direct access by company employees to potential customers via encrypted messaging platforms (e.g. WhatsApp, Zalo) [32, 33].

#### Blogs

Blogs are used to promote BMS, often as part of a broader, integrated digital media strategy [12, 13, 15, 17, 19•, 20–22, 34]. A content analysis of 719 unique blog posts that mentioned a BMS product found links to online retailers and discount coupons that trigger payments to the blogger [21].

Another content analysis of 59 blogs advertising and selling recipes and ingredients for women to make their own BMS at home found these sites did not contain statements expected on similar websites for commercial BMS; for example, 74.6% did not recommend consulting a healthcare provider before use, and 33.9% did not contain a statement on the superiority of breastfeeding [34].

One case report described strategic use of influencers to generate 350 blog posts promoting a new BMS product [35].

#### Websites

BMS manufacturers and retailers use websites to promote BMS products and brands [12, 16, 21, 22, 24••, 36–42]. One third of 27 Mexican websites held by six major BMS brands included sales promotions, and almost all of them solicited direct contact with parents (via email, live-chat or participation in contests) [16]. In the USA, BMS brand websites have been found to solicit user-generated photos and comments, provide educational content, offer access to infant feeding advice and provide tools to notify friends about products [12, 24••]. Websites held by BMS manufacturers and retailers in Asia offered sales incentives including cashback offers, iPhone giveaways, free shipping, online discounts, free samples, coupons and gifts [36].

Three papers reported that BMS manufacturer websites made health and nutrition claims for their products [13, 16, 41]. Analysis of websites and social media content in Mexico found most companies used images that idealised the use of BMS [16]. Two studies noted that the common practice of soliciting user-generated photos through contests allowed manufacturers to effectively circumvent proscriptions on the use of images idealising formula feeding [12, 21]. Four studies found that BMS company websites did not include satisfactory warning statements or disclaimers required by national legislation on their websites [21, 34, 39, 43].

Third-party websites are also used to promote BMS product and brands. One study found that online advertisements that promoted 11 BMS brands were viewed an average of 60.8 million times per month on third-party websites, chiefly on Amazon.com, facebook.com and Walmart.com [21].

Case reports describe the use of manufacturer and third-party websites by to increase engagement with BMS promotions and generate sales [44–48].

#### Digital Broadcast

Digital broadcasts (e.g. digital radio, podcasts) are used to promote BMS products and brands. An analysis of 504 h of digital television content in Thailand found that BMS advertisements were the most commonly occurring promotions [49].

Case reports also described the use of digital broadcast for BMS promotions. In Mexico, Nestlé used Spotify to stream short audio stories about babies’ development and invited users to unlock 30 min of uninterrupted listening by watching video advertisements for BMS products [50]. In China, a parenting video channel was created to rebuild confidence in BMS products after a major food safety scandal where melamine was detected in infant formula. Each of the 36 episodes featured questions from mothers on parenting topics, answered by nutritionists and experts from the BMS manufacturer itself, and the videos were viewed 13 million times in 3 months [51].

#### Direct Contact with Mothers

BMS companies make direct contact with mothers through social media posts and online messaging boards [12, 13, 16, 23•, 27]. In Mexico, nearly all 27 websites of six major BMS manufacturers included an invitation to contact (directly or indirectly) a company employee (email, chat) for advice on various parenting topics [16]. In South Africa, Nestlé used Facebook to invite mothers to a “Secret Mom’s Club” or join a WhatsApp group to win rewards [23•]. In Indonesia and Vietnam, Friso used follow-up conversations messaging app Zalo to convert parents to regular users (see Box 1) [33, 52].

#### Social Media Influencers

BMS companies provide financial or other incentives to expectant and new mothers to act as “influencers” by marketing their products to peers. An analysis of 719 blog posts found the majority were not identified as promotions or sponsored content. When this information was available, it was provided on separate pages (e.g. “about this blog”) [21]. BMS manufacturers use their social media accounts to establish financial relationships with mothers who then create testimonials about their experiences with BMS products [13]. Danone Nutricia paid a team of mothers to act as community managers and answer questions from fellow mothers on Facebook [22].

#### Providing Infant and Young Child Feeding Information

BMS manufacturers provide information about feeding infants and young children in several countries [9, 20, 22]. One study found that BMS manufacturers in South Africa provided information about infant and young child feeding to parents on Facebook [23•]. During the pandemic, BMS companies hosted educational webinars on topics related to COVID-19 and infant and young child feeding [25•]. In China, Yili partnered with a smart speaker manufacturer to create an artificial intelligence (AI) baby expert loaded with > 1000 mum-and-baby-related questions and answers (Q&A) to target young tech-savvy Chinese parents (see Box 1) [53], and Nestlé created a video channel for parents [51].

#### Data Mining, Segmentation and Targeting

Digital platforms generate massive datasets that can be used to promote BMS in new ways. It is virtually impossible for consumers to prevent collection of their data about pregnancy or infant caregiving [24••]. This is because data is routinely collected across devices (e.g. smartphones, tablets, smart watches) used by parents to access health information, connect with family and friends and research baby products. Activities like posting a pregnancy announcement on social media or making a search engine enquiry about pregnancy symptoms now trigger targeted advertising for baby products across a person’s digital devices. This kind of “cross-device tracking” remains largely unregulated [24••]. Another report described the way “social listening” is used to collect and analyse personal data to adjust campaigns to increase reach, engagement and ultimately sales of BMS [22].

Three publications highlighted how digital tools were used to collect personal data and analyse consumer preferences to facilitate targeted marketing of BMS [22, 24••, 28]. Manufacturers also reported using search engine optimisation to place their BMS brand websites first in response to relevant search terms [48].

Data can be collected from a variety of digital sources and used for various marketing purposes. BMS manufacturers and retailers collect consumer data themselves and may also purchase data and ad-targeting services from data brokers, who collect, maintain and manipulate information about consumers from online and offline sources (e.g. public birth records) [24••]. One study reported the use of user data to divide consumers into three distinct segments and to tailor a multi-faceted marketing effort to appeal to new parents according to the value they placed on ambition, happiness and safety [28]. Emerging tools such as Emotion Analytics and facial recognition may soon be used to market consumers depending on their mood [22].

BMS advertisers describe using predictive analytics, artificial intelligence (AI), machine learning and geo-targeting to identify customers. In China, Nestlé’s SuperNan combined social media data with artificial intelligence to create 51 different 30 s video advertisements targeted to seven audience segments based on specific allergies or sensitivities. The campaign garnered 450 million impressions, increased sales by 32% and increased unaided brand awareness [54].

In Indonesia, Danone used location data to link social media advertisements for Nutrilon growing-up milk with price discounts available in users’ immediate vicinity. Mothers received a link and were directed by Google Maps to nearby retail stores, increasing sales by 18% [55].

Analyses of BMS promotions on Facebook [12–17, 19•, 20, 22, 23•, 24••, 25•] consistently highlight its potential for complex, multi-level marketing. For example, manufacturers disseminate their own content (e.g. videos and posts) from company accounts and encourage users to engage with this content using the “like”, “share” and comment features. When users engage with promotional content, it is spread among their peers and appears on their own pages and in Facebook groups that provide opportunities for posting comments, questions and testimonials. All these user actions generate user data that can be used to generate insights about consumer behaviour. Facebook then harvests, analyses and sells user data to third parties (including BMS manufacturers) to facilitate further targeted advertising on its platform [24••].

### Exposure to Digital Marketing of BMS

In Australia, mothers reported using the Internet to access information on formula feeding (52%) and breastfeeding (73%) [40]. Hong Kong mothers ranked social media and online forums 6/8 of sources of health information on breastfeeding and infant feeding. They rated informational materials produced by BMS manufacturers high in attractiveness and accessibility but low in credibility compared to other sources [26].

In Ecuador, 18% of new mothers participated in industry-sponsored social groups that were mostly digital [20]. In Thailand, 83% of mothers reported seeing at least one BMS promotion in the past 6 months, over one quarter on social media or the Internet [20]. In Hong Kong, 56.8% of mothers surveyed reported always having encountered formula milk advertising on the Internet (less than television and radio (84.5%) but more than print media (52.8%) and point of sale (46.7%)) [26].

### Impact of Digital Marketing of BMS

Two studies reported an impact outcome. A longitudinal study of mothers in the USA found self-reported exposure to information about BMS online was associated with lower intention to breastfeed and lower breastfeeding initiation. Exposure to websites was more strongly associated with breastfeeding outcomes than exposure to traditional media (e.g. print and radio) [56]. Further analysis of these data found that pre-natal exposure to BMS marketing online increased brand switching among mothers who used infant formula [57].

## Discussion

There is evidence that digital marketing of BMS is occurring in a strategic and integrated fashion across a wide range of digital channels and platforms and that exposure to BMS marketing online is both common and harmful.

Strategies used by BMS manufacturers to market their products in digital environments include established marketing strategies adapted from traditional media. These include using images or text that idealises formula feeding; use prohibited health and nutrition claims in online content; offer coupons, samples and discounts through online retail; provide informational material on infant and young child feeding; and fail to provide prescribed text such as clear statements on the superiority of breastfeeding and that the product should only be used on the advice of a health worker.

Digital environments also offer advertisers additional tools for understanding, reaching and targeting pregnant women, mothers and young women based on their personal preferences. Large data sets fuel powerful algorithms that enable advertisers to identify pregnant women and mothers, generate insights into their values and concerns and deliver precisely targeted advertisements directly to their devices. BMS manufacturers can make direct-to-consumer contact through online messaging services that evade scrutiny from the general public and regulatory authorities. BMS brands commonly establish social groups to engage pregnant women and mothers on social media where they are encouraged to generate promotional content that influences their peers. Social media influencers (often parents themselves) are engaged to promote BMS products to their followers. There is some evidence that exposure to digital BMS promotions is associated with lower breastfeeding intention and initiation and that this association is stronger than seen with exposure to marketing in traditional media.

### Strengths and Limitations of the Review

We used a robust search strategy across multiple databases and used supplementary methods to identify further research. Overall, we used standard evidence synthesis methodologies and a transparent approach with the protocol registered a priori before data collection. Adding WARC case reports enabled development of a more comprehensive representation. We investigated case reports separately to demonstrate the transparency in our distinctive methodology.

### Implications for Research, Policy and Practice

Established strategies used by BMS manufacturers to market their products such as offering coupons, samples and discounts, providing informational material on feeding and using health and nutrition claims are clearly prohibited in the Code irrespective of where they occur. However, monitoring of digital marketing may be more difficult to conduct than traditional marketing when it is highly targeted and thus less visible to regulatory authorities. National regulations implementing the Code are also challenging to enforce when marketing originates across borders, thus requiring greater international cooperation. Development of automated monitoring tools and artificial intelligence could more routinely capture violations and support increased enforcement by relevant national authorities with appropriate sanctions.

Greater interpretive guidance is needed on the application of existing elements of the Code in a digital context. For example, Article 9’s provisions on labelling should apply to product descriptions provided in online retail listings as consumers cannot pick up product tins to read this information.

While the Code clearly prohibits the promotion of BMS without regard to media or methods, it does not directly address marketing strategies only made possible by the advent and popularity of digital media. The Code does not directly address the use of data analytics to generate marketing insights that inform marketing campaigns and facilitate delivery of tailored BMS promotions to precisely targeted audiences of pregnant women and mothers across multiple devices. It does not address the creation of social media clubs or parenting apps that facilitate further data mining and encourage consumers to generate promotional content. Lastly, the Code has nothing directly to say to, or about, social media influencers as a form of modern marketing personnel.

Social media platforms such as Facebook, Instagram, YouTube and Google can and do place voluntary restrictions on marketing content, including by preventing promotions with public health implications, including alcohol, weapons, pornography and foods that are high in fat, salt or sugar in some jurisdictions. However, as there are significant financial disincentives for platforms voluntarily closing established sources of marketing revenue, government-led regulatory solutions are also warranted.

Privacy legislation or other policies that protect consumers from the collection of their information across all digital channels and platforms may be helpful. In addition, regulatory innovation is needed to address incentives for influencers to promote BMS products. Concerns about these practices and the challenges of their regulation go far beyond the marketing of BMS specifically and create opportunity to work in coalition with a range of stakeholders interested more broadly in protection of consumer rights to achieve change.

## Conclusion

As consumers and parents spend an increasing proportion of their lives online, renewed action to ensure that regulation keeps up with constantly evolving and increasingly sophisticated digital marketing tactics will be critical to protect public health worldwide.

Addressing the widespread and harmful nature of digital marketing of BMS will require global action and stronger national measures to implement, monitor and enforce the Code. It may also require development or extension of policy guidance and regulation in new areas.

## Appendix Search Strategy

The following search strategy was used for PubMed, MEDLINE, Embase, APA PsycINFO and the Cochrane Central Register of Controlled Trials.

**Table:**

Concept	Search strategy (tested =)
1. Breast-milk substitute	“Infant Formula”[Mesh] OR “Bottle Feeding”[Mesh] OR “Milk Substitutes”[Mesh] OR ((“breast milk” OR “breast-milk”) AND “substitute*”) OR (“infant formula*” OR “baby formula” OR “formula-fed” OR “formula milk” OR “baby milk” OR “first milk” OR “baby formula” OR “artificial milk” OR “milk supplement” OR “supplemental feeding” OR “formula-supplement” OR “Breast milk substitute” OR “formula-feed” OR “formula feeding” OR “infant formula” OR “infant milk” OR “infant formula milk” OR “follow-on formula” OR “follow up formula” OR “transition formula” OR “toddler formula” OR “growing-up formula” OR “bottle feeding” OR “bottlefed”)
2. Digital marketing	“Marketing”[Mesh] OR “Social Marketing”[Mesh] OR “Direct-to-Consumer Advertising”[Mesh] OR “Internet”[Mesh] OR “Internet Use”[Mesh] OR “Internet-Based Intervention”[Mesh] OR “Electronic Mail”[Mesh] OR “internet” OR “website” OR “search engine” OR “SEO” e-mail OR “electronic mail” coupon OR console OR “online gam*” OR online OR internet OR digital OR “social media” OR “social network” OR “new media” OR advergam* OR Twitter OR tweet OR Instagram OR Weibo OR Wei-bo OR Youtube OR tiktok OR tik-tok OR facebook OR netflix OR “amazon prime” OR “over-the-top” OR streaming OR OTT OR blog* or influencer* OR SMS or “short messaging service” OR “podcast” OR “ app*” OR “online communit*”
3. Concepts add	Concept 1 AND 2

### World Advertising Resource Centre Database

We used a different strategy to locate WARC case reports given its different search functionality and use of keywords. We searched the WARC category of “baby food” for any item containing the words “formula” OR “milk” AND “digital”.

### Website Searches

Websites of the following organisations working to protect and promote full implementation of the Code were hand-searched to identify additional non-peer-reviewed research which met eligibility criteria:

- Save the Children Fund
- Global Breastfeeding Collective (WHO)
- World Breast Feeding Trends Initiative
- Baby Milk Action (IBFAN) and IBFAN International Code Documentation Centre
- Helen Keller International
- Changing Markets Foundation
- World Alliance for Breastfeeding Action
- Midwives Information & Resource Service
- International Confederation of Midwives (ICM)
- UNICEF

## References

[1] Global Breastfeeding Collective (2021). Global Breastfeeding Scorecard 2021.

[2] Piwoz EG, Huffman SL (2015). The impact of marketing of breast-milk substitutes on WHO-recommended breastfeeding practices. Food Nutr Bull..

[3] World Health Organization (1981). International code of the marketing of breast-milk substitutes.

[4] WHO and UNICEF (2018). Marketing of breast-milk substitutes: national implementation of the international code, status report 2018.

[5] WHO, UNICEF (2020). Marketing of breast-milk substitutes: national implementation of the international code, status report 2020.

[6] World Health Assembly. Fifty-Fourth World Health Assembly, WHA54.2 infant and young child nutrition; 2001.

[7] World Health Assembly. Seventy-Third World Health Assembly, WHA73(26) Maternal, infant and young child nutrition; 2020.

[8] American Marketing Association. What is marketing? American Marketing Association; 2017. https://www.ama.org/the-definition-of-marketing-what-is-marketing/, Accessed 2 May 2022.

[9] World Health Organization (2017). Guidance on ending the inappropriate promotion of foods for infants and young children implementation manual.

[10] World Health Assembly. Sixty-Ninth World Health Assembly, WHA69.9 Ending inappropriate promotion of foods for infants and young children; 2016.

[11] Nowell LS, Norris JM, White DE, Moules NJ (2017). Thematic analysis: striving to meet the trustworthiness criteria. Int J Qual Methods.

[12] Abrahams SW (2012). Milk and social media: online communities and the International Code of Marketing of Breast-milk Substitutes. J Hum Lact Off J Int Lact Consult Assoc.

[13] Senkal E, Yildiz S (2019). Violation of the international code of marketing of breastfeeding substitutes (WHO Code) by the formula companies via social media. Arch Dis Child.

[14] Vinje KH, Henjum S, Phan LTH, Nguyen TT, Mathisen R, Ribe LO (2017). Media audit reveals inappropriate promotion of products under the scope of the International Code of Marketing of Breast-milk Substitutes in South-East Asia. Public Health Nutr.

[15] Mejia P, Nixon L, Seklir L, Dorfman L, editors. Tweets, posts and health claims: a preliminary analysis of social media marketing of infant formula. In: APHA 2017 Annual Meeting & Expo (Nov 4-Nov 8). American Public Health Association; 2017.

[16] Lozada-Tequeanes AL, Hernández-Cordero S, Shamah-Levy T, Lozada-Tequeanes AL, Hernández-Cordero S, Shamah-Levy T (2020). Marketing of breast milk substitutes on the Internet and television in Mexico. J Paediatr Child Health.

[17] Mak SW (2015). Digitalised health, risk and motherhood: politics of infant feeding in post-colonial Hong Kong. Health Risk Soc.

[18] Walker M (2012). Stealth formula marketing—coming soon to a city near you?. J Hum Lact.

[19] • Mason F, Greer H. Don’t push it - why the formula milk industry should clean up its act. Save the Children International; 2018. **Holistic report on the current status and activities of the BMS industry and its key players, including role of social media and other digital strategies in promoting sales growth, with recommendations for what a variety of stakeholders can do to protect and promote breastfeeding.**

[20] Global Breastfeeding Collective. Marketing of breast milk substitutes: national implementation of the international code, status report. 2020.

[21] Harris JL, Fleming-Milici F, Frazier W, Haraghey K, Kalnova S, Romo-Palafox M (2017). Baby food facts: nutrition and marketing of baby and toddler food and drinks.

[22] Changing Markets Foundation. Milking it - how milk formula companies are putting profits before science. Changing Markets Foundation. 2017. https://changingmarkets.org/wp-content/uploads/2017/10/Milking-it-Final-report-CM.pdf. Accessed 2 May 2022.

[23] • Pereira-Kotze C, Doherty T, Swart EC. Use of social media platforms by manufacturers to market breast-milk substitutes in South Africa. BMJ Global Health. 2020;5(12):e003574. **Provides content analysis of recent digital marketing techniques in South Africa including invitations to secret mother groups and direct contact via WhatsApp and analyses their compliance with domestic legislation implementing the Code.** 10.1136/bmjgh-2020-003574 PMC7716659 33272942

[24] •• Wilking C. Reducing digital marketing of infant formulas. Public Health Advocacy Institute; 2020. **Focuses on how consumer data generated by expectant parents and infant caregivers is used to target them with digital marketing for BMS and provides legal and policy analysis of potential regulatory options to prevent this, particularly in the USA.**

[25] • Ching C, Zambrano P, Nguyen TT, Mathisen R, Tharaney M, Zafimanjaka MG. Old tricks, new opportunities: how companies violate the international code of marketing of breast-milk substitutes and undermine maternal and child health during the covid-19 pandemic. Int J Environ Res Public Health. 2021;18(5):1–29. **Provides content analysis of traditional and online marketing from 9 companies in 14 countries since 2020, systematically identifies key marketing themes and analyses how digital marketing in particular challenges implementation of the code.** 10.3390/ijerph18052381 PMC7967752 33804481

[26] Department of Health Hong Kong SAR Government. Survey on mothers’ views of formula milk promotion and information on infant and young child feeding. 2013. https://www.fhs.gov.hk/english/archive/files/reports/Survey_on_Mothers_views_on_FM_promotion_full_2016_final.pdf. Accessed 2 May 2022.

[27] Brewer BK, Andrzejewski C, Vij V, Crossley R, Kauer I. Methodology, results, and challenges in the assessment of baby food manufacturing companies' compliance with the International Code of Marketing of Breastmilk Substitutes and relevant national regulations in five countries. Matern Child Nutr. 2020;16.

[28] Hastings G, Angus K, Eadie D, Hunt K (2020). Selling second best: how infant formula marketing works. Glob Health..

[29] Zhao J, Li M, Freeman B (2019). A baby formula designed for Chinese babies: content analysis of milk formula advertisements on Chinese parenting apps. JMIR mHealth uHealth.

[30] Wyeth Nutrition. BabyNes for it moms. WARC Awards for Asian Strategy, Shortlisted: WARC; 2017.

[31] Wyeth Nutrition. Wyeth Gold: see the world at home. WARC Awards for Asian Strategy, Entrant: WARC; 2014.

[32] FrieslandCampina-Friso. FrieslandCampina Vietnam: because we care. MMA Smarties, Finalist, APAC: WARC; 2017.

[33] FrieslandCampina-Friso. Friso Indonesia: so you think you can grow. MMA Smarties, Gold, Indonesia: WARC; 2018.

[34] Davis SA, Knol LL, Crowe-White KM, Turner LW, McKinley E (2020). Homemade infant formula recipes may contain harmful ingredients: a quantitative content analysis of blogs. Public Health Nutr.

[35] Fonterra Brands Malaysia. Anmum Essential: mothers against secret sugars. WARC Awards for Asian Strategy, Highly Commended: WARC; 2012.

[36] International Baby Food Action Network (IBFAN), Breastfeeding Promotion Network of India (BPNI), IBFAN ICDC. Report on the monitoring of the code in 11 countries of Asia. 2018. https://www.bpni.org/wp-content/uploads/2018/12/Monitoring-of-the-Code-in-11-Countries-of-Asia.pdf. Accessed 2 May 2022.

[37] Bass J (2002). Webwatch. Br J Midwifery.

[38] Gunter B, Dickinson R, Matthews J, Cole J (2013). Formula manufacturers' web sites: are they really non-compliant advertisements?. Health Educ.

[39] Prado ISCF, Rinaldi AEM (2020). Compliance of infant formula promotion on websites of Brazilian manufacturers and drugstores. Rev Saude Publica.

[40] Newby R, Brodribb W, Ware RS, Davies PSW (2015). Antenatal information sources for maternal and infant diet. Breastfeed Rev.

[41] Berry NJ, Gribble KD (2017). Health and nutrition content claims on websites advertising infant formula available in Australia: a content analysis. Matern Child Nutr.

[42] Bartolini FLS, Do Amaral MDPH, Vilela MAP, De Mendonca A, Esther R, Vilela F (2009). Official monitoring of the Brazilian norm for commercialization of food for nursling and children of first infancy, rubber nipples, pacifiers, and nursing bottles – NBCAL. Braz J Pharm Sci.

[43] Silva KB, Oliveira MIC, Boccolini CS, Sally EDOF (2020). Illegal commercial promotion of products competing with breastfeeding. Rev Saude Publica.

[44] Illuma Organic. Illuma Organic: the green parents' data farm. Dragons of Asia, Best Digital Campaign, Bronze: WARC; 2018.

[45] Abbott Laboratories. Abbott Mommy Scoop. Dragons of Asia, best social media or word of mouth campaign, Bronze, Dragons of Malaysia: WARC; 2018.

[46] Danone-Nutrition. Nutrilon: capture every 7 days of Chinese mom. In: Han JWaI, editor. WARC Awards for Asian Strategy, Entrant: WARC; 2014.

[47] FrieslandCampina-Friso. Friso: winning over Vietnamese moms with a unique proposition. MMA Smarties, Bronze, Vietnam: WARC; 2019.

[48] Abbott Laboratories. Gain: the biggest GAINer Caravan - winning the approval of Asian tiger mothers. WARC Awards for Asian Strategy: WARC; 2013.

[49] Jaichuen N, evijvere S, Kelly B, Vongmongkol V, Phulkerd S, Tangcharoensathien V. Unhealthy food and non-alcoholic beverage advertising on children's, youth and family free-to-air and digital television programmes in Thailand. BMC Public Health. 2018;18(1):N.PAG-N.PAG. 10.1186/s12889-018-5675-3 PMC6003000 29902986

[50] Nestlé. Nestlé: Excella Gold Short Audio-Musical Stories in Spotify. MMA Smarties, Finalist, LATAM: WARC; 2018.

[51] Nestlé. Nestlé: NESLAC Experts talk. WARC Innovation Awards, Entrant, 2010: WARC; 2010.

[52] FrieslandCampina-Friso. Friso: so you think you can grow Vietnam. MMA Smarties, Gold, Vietnam: WARC; 2018.

[53] Yili. Jinlingguan: AI Baby Expert. WARC Awards for Asian Strategy, Entrant: WARC; 2020.

[54] SuperNAN: Super Relatable. WARC Media Awards, Shortlisted, Best Use of Data: WARC; 2020.

[55] Wulan AUK, Danone-Nutrition (2017). Nutrilon Footfall Measurement. WARC Media Awards, Entrant Effective Use of Tech.

[56] Zhang Y, Carlton E, Fein SB (2013). The association of prenatal media marketing exposure recall with breastfeeding intentions, initiation, and duration. J Hum Lact.

[57] Huang Y, Labiner-Wolfe J, Huang H, Choiniere CJ, Fein SB (2013). Association of health profession and direct-to-consumer marketing with infant formula choice and switching. Birth..

[58] Facebook for Business. Mead Johnson: increasing brand awareness with video ads on Facebook and Instagram. 2018. https://www.facebook.com/business/success/mead-johnson. Accessed 2 May 2022.

[59] FrieslandCampina-Friso. Friso Gold: to grow is to let go. In: Tran PT, editor. WARC Awards for Asian Strategy, Entrant: WARC; 2011.

[60] Nestlé. Nestlé: now everyone can breastfeed a child. WARC Awards for Asian Strategy, Entrant: WARC; 2016.

[61] Nestlé. Nestlé NAN Optipro 3: when the parents meet the experts. WARC Awards for Asian Strategy, Entrant: WARC; 2018.

[62] Nestlé. Nestlé: MOM & ME – a taste of pregnancy. In: Teo Hon Wui JN, editor. WARC Awards, Entrant, Effective Social Strategy: WARC; 2017.

[63] Nestlé. Nestlé NAN OPTIPRO 3: nurture your child. WARC Awards, Entrant, Best Use of Brand Purpose: WARC; 2017.

[64] Nestlé. Nestlé: NAN OPTIPRO Kid 4: celebrate christmas 2016. In: Teo Hon Wui JN, editor. WARC Awards, Entrant, Effective Content Strategy: WARC; 2017.

